# Values in Health Policy – A Concept Analysis

**DOI:** 10.15171/ijhpm.2016.102

**Published:** 2016-08-17

**Authors:** Lida Shams, Ali Akbari Sari, Shahram Yazdani

**Affiliations:** ^1^Department of Health Management and Economics, School of Public Health, Tehran University of Medical Sciences, Tehran, Iran.; ^2^Department of Medical Education, School of Medical Education, Shahid Beheshti University of Medical Sciences, Tehran, Iran.

**Keywords:** Values, Health, Policy-Making, Ideology, Principle, Belief

## Abstract

**Background:** Despite the significant role "values" play in decision-making no definition or attributes regarding the concept have been provided in health policy-making. This study aimed to clarify the defining attributes of a concept of value and its irrelevant structures in health policy-making. We anticipate our findings will help reduce the semantic ambiguities associated with the use of "values" and other concepts such as principles, criteria, attitudes, and beliefs.

**Methods:** An extensive search of literature was carried out using electronic data base and library. The overall search strategy yielded about 1540 articles and 450 additional records. Based on traditional qualitative research, studies were purposefully selected and the coding of articles continued until data saturation was reached. Accordingly, 31 articles, 2 books, and 5 other documents were selected for the review. We applied Walker and Avant’s method of concept analysis in studying the phenomenon. Definitions, applications, attributes, antecedents, and consequences of the concept of "value in health policy-making" were extracted. We also identified similarities and differences that exist between and within them.

**Results:** We identified eight major attributes of "value in health policy-making": ideological origin, affect one’s choices, more resistant to change over time, source of motivation, ability to sacrifice one’s interest, goal-oriented nature for community, trans-situational and subjectivity. Other features pinpointed include alternatives, antecedents, and consequences. Alternative, antecedents and consequences case may have more or fewer attributes or may lack one of these attributes and at the same time have other distinctive ones.

**Conclusion:** Despite the use of the value framework, ambiguities still persist in providing definition of the concept value in health policy-making. Understanding the concept of value in health policy-making may provide extra theoretical support to decision-makers in their policy-making process, to help avoid poor policy formulation and wastage of limited resources.

## Background

Decision-making is a highly value-laden process for which evidence cannot serve as the sole basis.^1^ Evidence and values both play important role at various levels of decision-making. While evidence shapes the decision-making process at the macro levels, values exert greater influence on decisions made. Values affect and shape initiatives at the macro level especially regarding which policies to be prioritized. However, at the clinical level, the role of values is reduced while the relevance of evidence is heightened during decision-making.^2,3^

Values are considered important component of policy-making and health system reforms.^4,5^ According to David Easton, politics are “authoritative allocation of values.”^6^ The role of values in policy-making goes back to the first decade of the 2000s. Advisers in America and Canada developed an explicit policy valuable framework for healthcare reform.^7^ There have been conflicts over the value concept since the time of Aristotle. Sociologists economists, political experts, and psychologists have given different interpretation to the concept of value.^8^ Values are not attached to a particular concept or discipline. In a broader scope, they are recognized as deep-rooted beliefs that affect objectives, decisions, behaviors,^7^ and policy implementation.^9^

Despite the existing evidence on the importance of value, stakeholders and decision-makers within the health sector have paid minimal attention to concept of value.^7,9^ For example, some believe that values are ethical principles, like equity and autonomy, while others interpret values as preferences. Besides, while some consider values to be collective beliefs, others argue them from the individuals’ perspective.^7^ McLaughlin considered values are preferences, needs, motivators, concepts, and situational needs.^10^ William referred to similar concepts and argued that values might be and closely related to concepts such as interests, pleasures, likes, duties, moral obligations, desires, wants, aversions, and attractions.^11^

Most people take values in their subjective assumptions without understanding their principal concepts, and they use them as their guidelines. As a result, there is lack of transparency regarding the definition of the term “value.” Until the substructures of value in health policy-making are meticulously analyzed, the value concept cannot be properly defined and utilized.^7^ Although, several studies have attempted to explore the concept of value and its ethical principles in clinical decisions.^3,12^ However, no study has explored the concept of “value in health policy.” Besides, given the obscurities which surround the definition of value, there is a need to make a clear distinction among the different the dimensions of the concept of value in this area. Until the elements of value in health policy-making are meticulously analyzed, the concept of value cannot be properly defined and utilized. Thus, This paper, therefore, attempts to illuminate the defining attributes of a concept of value and its irrelevant structures in health policy-making, by addressing the questions, (1) “What does the word ‘value’ mean in health policy-making? (2) What are the distinctions between values and other concepts such as principles, criteria, attitudes, and beliefs?” We anticipate our findings will help reduce the semantic ambiguities associated with the use of “values” and other concepts.

## Methods

The concept of value is semantically related to words such as belief, attitude, and principle, as such is often used inappropriately or interchangeably. This condition has created an ambiguity in the concept itself and in the method of analyzing it. In this study, a qualitative approach was applied and the views which distinguish the value concept from other similar or dissimilar ones are presented in this paper.^13^ Studies on concept analysis,^14,15^ either seek to clarify the vagueness associated with the concept or provide operational definition to the concepts.^16^ We applied Walker and Avant’s method of concept analysis in studying the phenomenon. The framework clearly and systematic stipulate the methods appropriate in achieving study objectives. The following 8 steps of concept analysis were followed; (1) First, is the selection of concept, (2) next, we determined the aims of the analysis, (3) we then identified all uses of the concept, (4) determined all defining attributes, (5) a model case was then constructed, (6) furthermore, we constructed borderline and contrary case, (7) antecedents and consequences were also identified, and (8) at the final stage we defined empirical referents.^17^ Since valid and credible instruments are needed to identify and determine empirical indices, the authors have a separate study in progress.

After identifying the concept, the most important step is to determine the scope and range of literature to be reviewed.^13^ An extensive search of literature (until May 2015) was carried out using PubMed, Embase, Elsevier, Emerlad, Scopus, Iran Medex, SID, Google Scholar, Google search engine and online dictionaries.

The following keywords were included in our search; principle, opinion, attitude, interest, belief, ethics, conviction, ideology, goals, criteria. These key terms were combined with the descriptor terms; policy-making, decision-making, and health policy. Reference lists and books were searched manually. We purposefully selected studies which were conceptually rich^18^ in definition and interpretations of the term value and its related concepts.^19^ Studies conducted in economics, clinical practice, marketing, and accounting were excluded. Our search was limited to studies published in English or Persian.

We searched for data until no new attribute for value in health policy and it related concepts were found in the literature. The overall search strategy yielded about 1540 articles and 450 documents. Studies were screened by reading the titles and abstracts of all records. Screening was done independently by two reviewers/authors (LS and SY). Disagreements between reviewers were discussed until an agreement was reached. Ultimately, 430 publications were selected. In the second phase, the studies were prioritized according to the conceptual richness of finding studies and documents and were studied.^18^ Based on the qualitative research approach, data collection continued until the basic elements of the study were saturated. So that, no new attribute for value and related concepts in health policy was found in the literature. Finally, 2 books, 31 articles, and 5 additional records emerged to be conceptually rich in definition and interpretation of the term value and its related concept.

Coding was done manually using the method of concept analysis. After responses to the research question and the specified attributes of the value concept and other related concepts were gathered, the necessary tests and the adequacy test were completed by several health policy experts. Those necessary tests were conducted to assess conceptual attributes so that the necessary specified attribute came to be among the features of the concept. The adequacy test used to evaluate this issue dealt with whether the same characteristics used to define the concept have an adequate number of quartiles or if they need more features to clearly define the value concept.^13^

## Results

### Defining Attributes of the Value Concept in Health Policy-Making

Determining operational definition of attributes are critical in differentiating one concept from other related concepts.^13^ Values are special form of beliefs^17^ which guides people decisions, like a map.^6,20-25^ They are the basis of attitudes, motives, behaviors, and basic assumptions underlying the existence of society. Thus, values are abstract, internal, and subjective concepts.^6,26,27^ Values are of different classification including terminal values. Values-based policies are contingent on terminal values. Terminal values indicate the final results and outcomes of policy-making.^24,25,28^ These values are made up of eight major attributes. Thus, terminal values should:

- Be based on ideologies.^29,30^
- Affect one’s choices.^5,6,26^
- Be more resistant to change over time than other form of beliefs.^9,31-35^
- Serve as a source of motivation for people.^36^
- Be of great significance^6^ that people are ready to bear difficulties or sacrifice their interests to realize them.^37^
- Not be restricted to a specific situation (trans-situational).^9,24,25^
- Be goal-oriented nature for people and society.
- It is more abstract compared to other concepts.^6,8,26,27^

So, the concept of value cannot be attributed to health policy in the absence of any of these attributes.

### Building the Model Case

Model case and analysis portrays real example of a concept in the outside world by defining the attributes of concepts. It is a way of providing better understanding of a concept under study.^17^

*Model case: The moral theory governing health policy-making in country A is “egalitarian liberal.” The belief of the country is that this ideology could promote human survival (attribute 1). Values such as social equity, social solidarity, and sustainable development are stressed in the country’s higher order documents. In the higher order documents of the Ministry of Health and Medical Education (MoHME), values such as health equity, social solidarity in health, and health improvement are considered as dominant drivers of the health system (attributes 3 and 7). According to the governing theory, the most important values are to ensure that the less privileged populations are able to utilize health services. As a result, people are willing to ignore other values or to consider other values least important in order to fulfill what is relevant (attribute 5). Political parties also hold a firm belief in values of the Ministry of Health (MoH) and even use them in their campaign slogans (attribute 4). These values serves as references during decision-making in different areas of health systems, including human resources, allocation of financial resources, payment systems for providers, legislation, etc. (attribute 6). Given the abstract nature of the concept of value, experts within the health sector are obliged to provide a substantial interpretation of the concept (attribute 8). “Equitable distribution of health benefits” emerged as the main criterion for setting the priority of services (attribute 2).*

### Definition of Alternatives

Alternative cases include concepts that have a lot in common in relation to a particular concept. In such instances, alternative cases may be used in replace of the original concept due to their nominal or content similarities, although they might not necessarily convey the same meaning as the original concept.^38^ These alternatives may include borderline**,** contrary, and related cases, etc.

*Borderline cases* contain some of the vital attributes of a concept not all of them.^14^ They are very similar to model case but distinctive from other related case models. Identification of borderline cases can help clarify basic attributes of case model, and significantly reduces borderline ambiguities.^17^

Certain values play an intermediary role and serve as means to achieve terminal values and, are termed as *instrumental values.*^24,25,28^ They usually emanate from health system strategies. Terminal and instrumental values are both considered *content values*. That means their realization should be sought in the implementation of policy options. The successful development, approval, and implementation of policies require initially instrumental values and then terminal values.

Value-based policy-making is not only limited to policy options and how they are chosen, but also during the policy-making process the agreed values must be respected. Thus, the realization of *process values* is meaningful only during the process of policy-making^39^ or policy implementation. Focusing on the *policy-making process values* guarantees the successful development and approval of policies, and focusing on the *implementation process values* guarantees the successful implementation of policies. As illustrated, attributes of instrumental and process values differ from that of terminal values. Besides, several terminal values do have either lesser or no degree of attributes (Table 1).

**Table 1:** Differences Between Modal Case and Borderline Cases

**Attribute**	**Borderline Cases**			
	**Content Values**		**Process Values**	
	**Terminal Values**	**Instrumental Values**	**Policy-Making Values**	**Implementation Values**
Definitional attribute				
Be based on ideologies	‏***	‏**		
Affect one’s choices	‏***	‏***	‏***	‏***
Be more resistant to change over time than other form of beliefs	‏***	**	‏*	‏*
Serve as a source of motivation for people	‏***			
Be of great significance that people are ready to bear difficulties or sacrifice their interests to realize them	‏***	**		
Not be restricted to a specific situation (trans-situational)	‏***		‏***	‏***
Be goal-oriented nature for people and society	‏***	*		
It is more abstract compared to other concepts	‏****	‏***	**	**
Discriminate attributes				
Practical importance for realization of goals		‏***		
Guaranteed successful development and approval of policies			‏***	
Guaranteed successful implementation of policies				‏***

***High; ** Low; *Very lows; No mark: lacks the attribute.

*Borderline case: The most important value in the health system of country A is ensuring that the less privileged populations enjoy health services (terminal values). Therefore, values including improving access to health services, responding to people’s needs, and improving service quality (instrumental values) remain priorities within the health services to achieve terminal values.*

*On the other hand, values of feasibility, stakeholders’ approval, and cost-effectiveness were underscored during the process of policy-making. The implementation process values include transparency, evidence, and participation of people.*

*Contrary cases* do not have the attributes of the value concept. Besides, they specify what the concept is not made of, and easy to identify the concept under question.^13^ Moreover, contrary cases do not add any new information for analysis.^40^ Therefore, any concept that lacks the attributes below will have no value concept in health policy-making.

- Should be a subcategory of beliefs.
- Affects one’s choices.
- More resistance to change over time compared to other beliefs.
- Evokes excitement in people.
- Trans-situational.
- Goal-oriented nature for people and society.

*Contrary case: In country B, a larger proportion of decisions at the policy-making levels are based on immediate intuitions, individual preferences, and past experiences of policy-makers without any emphasis on the reference values of the system of health policy-making. As a result the decision regarding prioritization, acceptability, desirability, and sufficiency of macro-policies are either based on personal opinions of policy-makers or evidence/findings which satisfy the beliefs of the policy-makers. Therefore, health policies are amended when mangers leaves office or when their personal beliefs are base of decision-making, resulting to ineffectiveness, inefficiency, inequity, and dissatisfaction within the health sector.*

*Related cases* are cases that are connected to a concept but do not contain the fundamental attributes. However, related concepts often lead to uncertainties and inaccuracy about the concept.^14^ “Principles and criteria” are example of related cases in health policy-making.

*Principles* are fixed, objective, external, directional, self-evident and self-validating truths that always show the direction like a compass.^41^ As for Coming from different sources, they act as rules of thumb for policy-makers during the policy-making process. For example, scientific principles are instrumental interventions that have been proven effective in directing policy-makers towards valid and reliable terminal values. While juridical principles originate from laws and regulations, moral principles form part of such principles and govern the decision-making systems of a country.^42^ Unlike values which indirectly affect decisions, through a criterion system and in an analytical framework, the effects of principles on decisions are usually direct and non analytical. That is why values are compared to a map and principles to a compass. In other words, attention to principles can lead to the early rejection of some policy options so that policies that comply with the principles can be assessed against the criterion or standards. In terms of objectivity, principles lie between variables, criteria, and content values (Table 2).

**Table 2:** Differences Between Modal Cases and Related Cases - Principles

**Attribute**	**Related Cases**	
	**Terminal Values**	**Principles**
Definitional attribute		
Be based on ideologies	‏***	
Affect one’s choices	‏***	‏***
Be more resistant to change over time than other form of beliefs	‏***	
Serve as a source of motivation for people	‏***	
Be of great significance that people are ready to bear difficulties or sacrifice their interests to realize them	‏***	
Not be restricted to a specific situation (trans-situational)	‏***	
Be goal-oriented nature for people and society	‏***	
It is more abstract compared to other concepts	‏***	*
Discriminate attributes		
Direct effect on decision-making		‏***
Related to a particular domain of the health system		‏***

***High; ** Low; *Very lows; No mark: lacks the attribute.

*Related case: In the health system of country A, and according to the World Health Organization Declaration of Alma-Ata1978, a primary healthcare system (PHC) was the key to achieving health goals for everyone by the year 2000. This they anticipate can help improve equity in the health system. Therefore, any proposed plan or service provision reforms by the MoH should include the principles of PHC.*

*Criteria* are measurable concepts worthy of value judgments and serve as basis for decision-making.^43-45^ Generally, the concepts of values are abstract in nature^21^ and plays challenging role in the decision-making process. As a result criteria are selected as intermediary concepts between values and decision-making. In other words, value judgments about different policy options are based on the scores of each option obtained from different criteria (Table 3).

**Table 3:** Differences Between Modal Cases and Related Cases - Criteria

**Attribute**	**Related Cases**	
	**Terminal Values**	**Criteria**
Definitional attribute		
Be based on ideologies	‏***	‏*
Affect one’s choices	‏***	‏***
Be more resistant to change over time than other form of beliefs	‏***	
Serve as a source of motivation for people	‏***	
Be of great significance that people are ready to bear difficulties or sacrifice their interests to realize them	‏***	
Not be restricted to a specific situation (trans-situational)	‏***	
Be goal-oriented nature for people and society	‏***	
It is more abstract compared to other concepts	‏***	
Discriminate attributes		
Measurability		‏***
Related to a particular domain of the policy-making system		‏***
Directly affects decision-making		‏***

***High; ** Low; *Very lows; No mark: lacks the attribute.

*Related case: The terminal value governing the system of health policy-making in country A is “to ensure that the less privileged populations are able to utilize health services.” The instrumental values for realizing this value include; improving access to health services, responding to people’s needs, and improving service quality of priority health needs. Health system experts allocate resources on the basis of criteria such as population, disease burden, social and economic status of the population.*

### Antecedents and Consequences

*Antecedents* are events that exist prior to the occurrence of a concept. Yet, they cannot be considered to be similar to a “cause.”^19^ In values-based policy-making, beliefs are antecedents of terminal values.

Beliefs are part of human convictions (confirmation of a subject by reason^43^). There are two types of human convictions - justified and unjustified conviction. Beliefs are unjustified convictions (its accuracy need not be justified by reasoning or evidence). On the other hand, knowledge is a part of human conviction of which its accuracy should be confirmed either by empirical evidence or reasoning - justified conviction.

In the words of Aristotle, knowledge is a “justified true belief.”^46^ Since belief is defined as unjustified conviction,^47^ the term “justified true conviction” has been used to define knowledge.

*Antecedents: There was a general conviction among health experts in 2002 that other provider payment mechanisms have advantages over fee-for-service payment system (belief), but no systematic review has ever proven this advantage (scientific belief). So, this conviction is supported without any scientific evidence*.

*Consequences* are events that happen after an occurrence.^14^ In general, terminal values lead to a change in behavior or action. Change in behavior can be implicit or explicit. Implicit path affects the attitudes of decision-makers and experts (Figure). Attitude is a positive or negative emotional state toward a particular subject. It is a reflection of how one feels about an object or person.^48^

**Figure:**
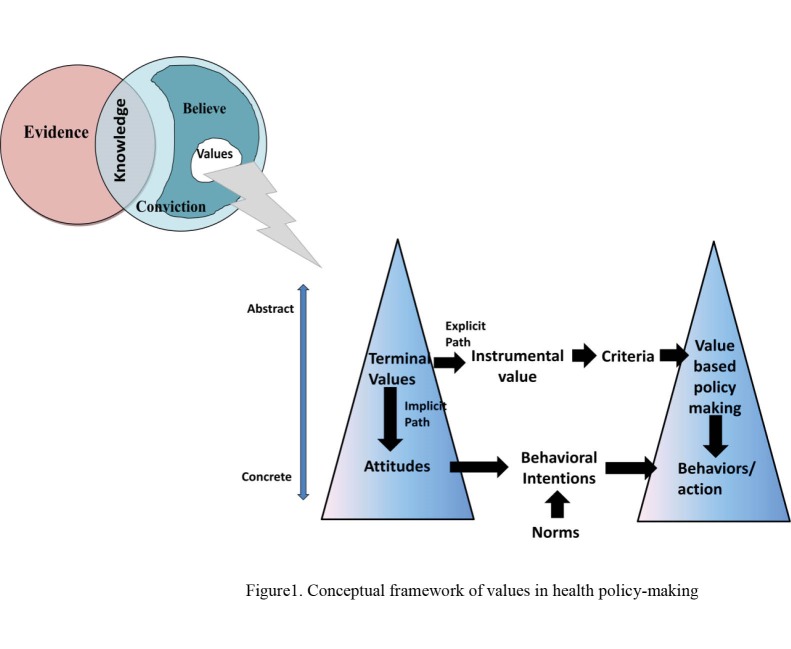
Conceptual Framework of Values in Health Policy-Making.

Terminal values explicitly lead to values-based policy-making by explaining the value systems, and linking it with the instrumental values and a criterion system. Values-based policy-making is a form of policy-making where relationships between values and policy options and options appraisal mechanisms are completely transparent and well-defined (Table 4).^49-51^

**Table 4:** Differences Between Modal Cases and Consequences

**Attribute**	**Consequences**	
	**Terminal Values**	**Attitudes**
Definitional attribute		
Be based on ideologies	‏***	
Affect one’s choices	‏***	‏***
Be more resistant to change over time than other form of beliefs	‏***	‏*
Serve as a source of motivation for people	‏***	‏*
Be of great significance that people are ready to bear difficulties or sacrifice their interests to realize them	‏***	‏*
Not be restricted to a specific situation (trans-situational)	‏***	
Be goal-oriented nature for people and society	‏***	
It is more abstract compared to other concepts	‏***	‏*
Discriminate attributes		
Emotional state toward a particular subject		‏***

***High; ** Low; *Very lows; No mark: lacks the attribute.

*Consequence: The terminal value governing the system of health policy-making in country A is “to ensure that the less privileged populations are able to utilize health services.” The instrumental value for realizing this value is by improving access to health services and responding to people’s needs. As a result, policy-makers consider certain criteria - including the burden of disease and social and economic status of the population in allocation of resources (values-based policy-making).*

*In the health system of country C, since the majority of policy-makers are Shiite Muslims, health equity as a fundamental value has a sublime place in the value framework of policy-makers. This makes them to support implicitly equity-oriented initiatives (attitude).*

## Discussion

The application of Walker and Avant’s model provided a systematic approach to identify the concept of value in health policy-making. It also helped clear the vagueness associated with the concept by presenting eight key attributes, and specifying the distinctions between value concept and other model cases - ie, related cases, borderline, and contrary cases. Due to the ambiguities inherent in defining the concept of value in health policy-making, few questions have so far been correctly answered. They include “What values are health policies based on?” or “Do the health policies observe those values?”

Various researchers and authors, across several disciplines – eg, economic, psychology, and clinical practice have provided different definition of the concept “value.”^8,52^ To the psychologist, the concept of value is generally considered as the model of selective orientations^11^ which relates to individuals preferences, motives, needs, and attitudes.^10^ Sociologists also interpret value to mean social concepts including norms, ideology, and commitments. The word “value” has also been widely used by *Economist* in discussing the concept of utility, trade, and price.^26^ Also, Sackett et al from the clinical point of view defined the concept of value as “…the unique preferences, concerns, and expectations each patient brings to a clinical encounter and which must be integrated into clinical decisions if they are to serve the patient.”^12^

According to Schwartz, “values are trans-situational goals which guide the principles of individuals or other social institutions.”^53^ Brown thought of “values as morals, beliefs, conduct, and qualities of people and groups.”^8^ Hofstede interpreted “values as the tendency to prefer something over another.”^31^ Smith considered values to be attributes of the world in relation to its people and of people in relation to the government shaping the values.^54^ Deth and Scarbrough stated that values are non-empirical and internal. They are concepts which engage moral dialogue in choosing what is favorable.^26^

McLaughlin thought of values as preferences, needs, motivators, concepts, and situational needs.^10^ William provided a long list of value-related concepts and argued that values might be closely related to concepts such as interests, pleasures, likes, preferences, duties, moral obligations, desires, wants, needs, aversions, and attractions.^11^

Based on the definition provided by Woodruff and Divesta, values are general life conditions which can have an impact on one’s welfare.^55^ Similarly, Nye suggested that “values are the most abstract concept which encompasses general sets of goals, feelings, and experiences.”^26^

In the political sciences, the value concept is at the heart of Easton’s definition of politics as, “interactions through which values are allocated for a society.”^56^

Analysis showed that the main attributes of value in health policy-making are ideological origin, affect one’s choices, more resistant to change over time, source of motivation, ability to sacrifice one’s interest, goal-oriented nature for community, trans-situational and subjectivity. Alternative, antecedents and consequences case may have more or fewer attributes or may lack one of these attributes and at the same time have other distinctive ones.

Terminal values such as justice, health, and satisfaction require series of instrumental values such as efficiency, quality, availability, and effectiveness. Nevertheless, the importance of each instrumental value for the achieving of each terminal value is different.

Numerous studies, have highlighted on the concept of value and its related dimensions.^7,9,25,26,31,35,36,54,55,57^ Some studies discussed some of the differences or similarities between values and other concepts.^10,55^ Yet, none have attempted to spell out the concept of value within the context of health policy-making.

## Conclusion

Despite the use of the value framework, ambiguities still persist in providing definition of the concept value in health policy-making. This study presents attributes from the health literature and provides list of values relevant to health policy-making to help prevent structural discrepancies between the concept of value and other related concepts such as principles and criteria.

Having the value concept clarified in health policy-making could pave the way for theoretical expansion and execution of value so that the mere adherence to evidence would no longer be the basis for decision-making. The ambiguity of concepts can cause poor policy formulation and wastage of limited resources.

## Acknowledgements

This work was part of a PhD dissertation, funded and financially supported by the Tehran University of Medical Sciences, Tehran, Iran. The authors would like to thank Mr. Abraha Woldemichael for paper edition and Mr. Taha Nasiri and Mr. Ebrahim Saadatjoo for searching publications that are helpful for conceptualizing this research.

## Ethical issues

No applicable.

## Competing interests

The authors declare that they have no competing interests.

## Authors’ contributions

Study design: LS, SY, and AAS; Acquisition of evidence: LS, SY; Interpreting the findings: LS, SY, and AAS; Technical support: SY and AAS; Drafting of manuscript: LS, SY, and AAS.

## Authors’ affiliations

^1^Department of Health Management and Economics, School of Public Health, Tehran University of Medical Sciences, Tehran, Iran. ^2^Department of Medical Education, School of Medical Education, Shahid Beheshti University of Medical Sciences, Tehran, Iran.
